# The role of vitamin D in pediatric systemic lupus erythematosus - a double pawn in the immune and microbial balance

**DOI:** 10.3389/fimmu.2024.1373904

**Published:** 2024-04-23

**Authors:** Vasile Valeriu Lupu, Ancuta Lupu, Elena Jechel, Iuliana Magdalena Starcea, Gabriela Stoleriu, Ileana Ioniuc, Alice Azoicai, Ciprian Danielescu, Anton Knieling, Reka Borka-Balas, Delia Lidia Salaru, Ninel Revenco, Silvia Fotea

**Affiliations:** ^1^ Pediatrics, “Grigore T. Popa” University of Medicine and Pharmacy, Iasi, Romania; ^2^ Clinical Medical Department, Faculty of Medicine and Pharmacy, “Dunarea de Jos” University of Galati, Galati, Romania; ^3^ Faculty of Medicine, “Grigore T. Popa” University of Medicine and Pharmacy, Iasi, Romania; ^4^ Pediatrics, “George Emil Palade” University of Medicine, Pharmacy, Science and Technology, Targu Mures, Romania; ^5^ Pediatrics, “Nicolae Testemitanu” State University of Medicine and Pharmacy, Chisinau, Moldova

**Keywords:** pediatric systemic lupus erythematosus, vitamin D, innate immunity, adaptive immunity, diet, microbiota, individualized therapy

## Abstract

Having increased popularity during the Covid-19 pandemic, vitamin D3 is currently impressing thanks to the numerous researches aimed at its interactions with the body’s homeostasis. At the same time, there is a peak in terms of recommendations for supplementation with it. Some of the studies focus on the link between autoimmune diseases and nutritional deficiencies, especially vitamin D3. Since the specialized literature aimed at children (patients between 0-18 years old) is far from equal to the informational diversity of the adult-centered branch, this review aims to bring up to date the relationship between the microbial and nutritional balance and the activity of pediatric systemic lupus erythematosus (pSLE). The desired practical purpose resides in a better understanding and an adequate, individualized management of the affected persons to reduce morbidity. The center of the summary is to establish the impact of hypovitaminosis D in the development and evolution of pediatric lupus erythematosus. We will address aspects related to the two entities of the impact played by vitamin D3 in the pathophysiological cascade of lupus, but also the risk of toxicity and its effects when the deficiency is over supplemented (hypervitaminosis D). We will debate the relationship of hypovitaminosis D with the modulation of immune function, the potentiation of inflammatory processes, the increase of oxidative stress, the perfusion of cognitive brain areas, the seasonal incidence of SLE and its severity. Finally, we review current knowledge, post-pandemic, regarding the hypovitaminosis D – pSLE relationship.

## 1 Introduction

Pediatric systemic lupus erythematosus (pSLE) is an autoimmune condition, which gathers under its umbrella multiple pathognomonic clinical and paraclinical disturbances. It has a still incompletely elucidated pathogenesis, based on environmental, hormonal and genetic sensitization factors. Pediatric systemic lupus erythematosus broadly follows the diagnostic and management criteria found in adults. The difference between the two lies in the aggressiveness of symptoms among children and adolescents compared to adults. Also, atypical forms of pSLE can be registered, burdened by a severe prognosis, when the onset is under 5 years of age (1, 2). At the immunological level, it is characterized by the production of autoantibodies, the activation of the complement system and immune deposits. Genetically, up to 7% of pSLE patients are estimated to have monogenic disorders, while in the remainder of the affected population genetic variability overlaps with additional environmental factors (2).

Recent controversies regarding the management possibilities of systemic lupus erythematosus (SLE) concern tolerogenic drugs. Among these we mention the lupuzor. Its biochemical component is represented by a small fragment of phosphorylated ribonucleoprotein in the 140-serine position. In the immunological dynamics of SLE, it seems that lupuzor favors the expansion of regulatory T cells to the detriment of effector ones. As a result, the lupuzor restores tolerance to nuclear autoantigens (3). A similar effect is observed in the case of vitamin D3 deficiency correction. Vitamin D3 deficiency has been linked to increased C-reactive protein (CRP), hyperlipidemia, insulin resistance, blood flow changes, and atherosclerosis. Correction of hypovitaminosis may also reduce cardiovascular risk and SLE activity. However, caution is required because of the risk of worsening lupus nephritis (3–5). Vitamin D3 is part of the group of fat-soluble secosteroid vitamins, having numerous implications in the body, among which we mention its role in cell growth, phospho-calcium balance, neuro-muscular activity and immunity. It is found in low quantities in patients with SLE despite exposure to the sun throughout the year. In this sense, it performs its function by reducing phagocytosis and the activity of the major histocompatibility complex, in parallel with favoring the maturation of natural killer cells intended to balance the balance of immune tolerance (4, 6, 7).

Analyzing the specialized literature from the last decade, we found a strong correlation and an increased interest in studying the microbial balance, nutritional components and how they are reflected in dictating the appearance or evolution of various systemic pathologies. Considering our scientific orientation towards pediatrics, induced by its current practice, we considered it appropriate to carry out a narrative review using international databases to identify relevant articles related to the impact of intestinal dysbiosis and nutritional imbalances among children with systemic lupus erythematosus. Searches focused on words and key phrases commonly used to describe pSLE, nutritional deficiencies, and their main lines of support and treatment (e.g., pediatric systemic lupus erythematosus, autoimmune disease, disease of a thousand girls, nutritional disorders, poor diet, dysbiosis, paraclinical disorders, adjuvant means). We have added to these terms useful in finding information about SLE pathogenesis and shadow pathophysiological mechanisms (e.g., autoimmunity, risk factors, innate immunity, adaptive immunity, systemic inflammation, dendritic cells, intestinal permeability, bacterial translocation, dysbiosis). The crossroads of the searches coincided with the impact of hypovitaminosis D during the pandemic for pSLE patients.

The main objectives of the work were the dissemination of general knowledge regarding the factors that predispose to increase the susceptibility to develop pSLE. To these is added the desire to deepen the main links between the patho-physiological mechanisms of the disease. The desired practical purpose resides in a better understanding and an adequate, individualized management of the affected persons. We focus on both the acute episode and the disease-free interval in order to reduce morbidity, especially in the pediatric population known as a vulnerable population.

## 2 Epidemiology

It is estimated that, of the total number of SLE cases encountered globally, pSLE represents approximately 15-20%. The average age of diagnosis is 12 years, the gender ratio remains between 2.3-9:1 in favor of girls (1). Regarding the incidence and prevalence of the pathology, they are between 0.36-2.5, respectively 1.89-34.1 per 100,000. The mortality rate reported in comparison with the general population is increased among patients with SLE, reaching up to three times in the case of those with pSLE (2). The genetic predisposition towards the development of the condition is argued by the existence of a concordance of 25% in monozygotic twin pairs, compared to 2% in dizygotic ones (8).

Ethnic variability is well represented within this pathology, with the reported frequency being high in Asians, African Americans, Hispanics and native Americans. The literature notes a more typical presentation, accompanied by a more pronounced impairment of renal function and an increased therapeutic need, among minority populations (e.g., black subjects from Africa/Caribbean), compared to Caucasians (2, 9). The risk of progression of cutaneous lupus in the form of systemic damage is also recorded, ranging between 0-31% among children (as opposed to adults where the percentage increases up to 42%). The risk factors that can anticipate this outcome are the positivity of antinuclear antibodies, hematological abnormalities and a high diagnostic score at the time of the initial evaluation (10).

Despite the decreasing incidence of diseases that directly target 25-hydroxyvitamin D (25(OH)D) deficiency, such as rickets and osteomalacia, its values remain below the normal threshold in approximately 1 billion people. In percentage reports, it is estimated that approximately 40% of the European population has 25(OH)D deficiency/insufficiency (< 30 ng/ml). 13% of them fall within the severe deficiency limits (< 12 ng/ml). The risk factors that lead to the disruption of their metabolism are represented by insufficient exposure to sunlight, inadequate food sources, malabsorption caused by gastrointestinal diseases or, more recently, obesity. The latter seems to influence the serum level of 25(OH)D by sequestering it in the adipose mass (7, 11, 12). The effects of 25(OH)D deficiency can be felt from early childhood and can affect all stages of life. A brief list of pathologies in which calcifediol plays an important role can be opened with the well-known rickets, osteomalacia, osteoporosis and culminating with intrauterine growth restrictions, musculoskeletal, cardiovascular, neuro-degenerative, autoimmune, diabetes or gestational diabetes, fertility, preeclampsia or even cell proliferation and malignancies (13). Although it is not a universal panacea, supplementing 25(OH)D deficiencies can be effective where they exist, studies in the literature demonstrating the correlation between its low level and proteinuria, with the detection of vitamin D3 binding protein in the urine among patients with SLE (14).

## 3 General considerations regarding bioclinical aspects suggestive of pSLE

In order to optimally frame and evaluate patients with SLE, **Table 1** summarizes the main lines of diagnosis and classification of the pathology according to the degree of involvement. Massias JS. et al. points out that the clinical-biological parameters and the severity of the condition are inconstant in different age groups, respectively the pre- (≤7) and peripubertal (8-13) and adolescent (14-18) periods (17). However, the main difference is between lupus in adults and the pediatric form. Here, the juvenile form exhibits greater disease activity, thus causing aggression and an increased pharmacological burden. Clinically, the main concerns are increased morbidity and mortality (partially drug-induced), a more florid skin manifestation at the time of diagnosis and more severe organ damage. Among the affected systems, we preferentially retain the cardio-vascular, neurological and renal systems (2).

**Table 1:** Clinical-biological parameters and severity in SLE (adapted from Levy DM. et al., Trindade VC. et al. and Fava A. et al.) (9, 15, 16).

Type	Parameters
Clinical	* general symptoms - fever, lymphadenopathy, weight loss * malar rash - “butterfly”, erythematous, non-pruritic, which heals without scarring * rarely discoid rash * photosensitivity * alopecia * Raynaud’s phenomenon * livedo reticularis * vasculitis/petechiae/purpura * oral/nasal/digital ulcers * non-erosive and non-deforming arthritis, arthralgias, avascular necrosis, bone fragility * serosity * neurological damage – convulsions/psychosis, after elimination of other causes, aseptic meningitis, neuropathies, mood disorders, headaches, cognitive dysfunctions
Biology	✔ Kidney damage – proteinuria >0.5 g/day or cellular casts – lupus nephritis - divided into 5 classes (assessment requires biopsy) ✔ Hematological impairment – mild leukopenia: 3,000 – 4,000/mm3 – lymphopenia: <1500 cells/mm3 – anemia – mild/profound thrombocytopenia <150,000 - 10,000 – antiphospholipid antibodies ✔ Immunological disorders – ANA – high sensitivity – anti-dsDNA – high specificity – anti-Ro (anti-SSA) and anti-La (anti-SSB) antibodies – anti-SM, anti-RNP, ENA – hypocomplementemia (C3,C4) + discordant ESR – CRP
Severity score (SDI)	→ It reflects the activity of the disease by measuring the systemic damage (ocular, neurological, renal, pulmonary, cardiovascular, gastrointestinal, musculoskeletal, cutaneous, metabolic, oncological and peripheral vascularization), gonadal insufficiency, growth restriction or delay in the appearance of the characters secondary sex → It can be influenced by steroids, immunosuppressive therapy or biological agents → It is recommended to be evaluated annually

ANA, antinuclear antibodies; anti-dsDNA, anti-double-stranded DNA antibodies; anti-SM, anti-Smith antibodies; anti-RNP, anti-ribonuclear proteins; ENA, extractable nuclear antigens; anti SSA, Sjögren syndrome A antigen; anti SSB, Sjögren syndrome B antigen; ESR, erythrocyte sedimentation rate; CRP, C reactive protein; SDI, Systemic Lupus International Collaborating Clinics/American College of Rheumatology Damage Index score.

The main diagnostic criteria in pSLE are therefore based on the well-known Systemic Lupus International Collaborating Clinics (SLICC) criteria, used among adults. Referral to the specialist is recommended when we encounter the coexistence of two SLICC criteria, doubled by the positivity of antinuclear antibody (ANA) antibodies. Alternatively, it can be discussed when there is a SLICC criterion, a clinical criterion and positive ANA antibodies (18). The SLICC criteria include clinical (malar or discoid eruption, photosensitivity, oral ulcers, non-scarring alopecia), biological (anemia, lymphopenia, thrombocytopenia, leukocytosis, low complement C3 and C4 fractions, antiphospholipid antibodies, ANA, anti-double-stranded deoxyribonucleic acid antibodies [anti-dsDNA] and anti- anti-Smith (SM) positive) and systemic manifestations (arthritis, serositis, renal/neurological lesions) (19).

**Figure 1:**
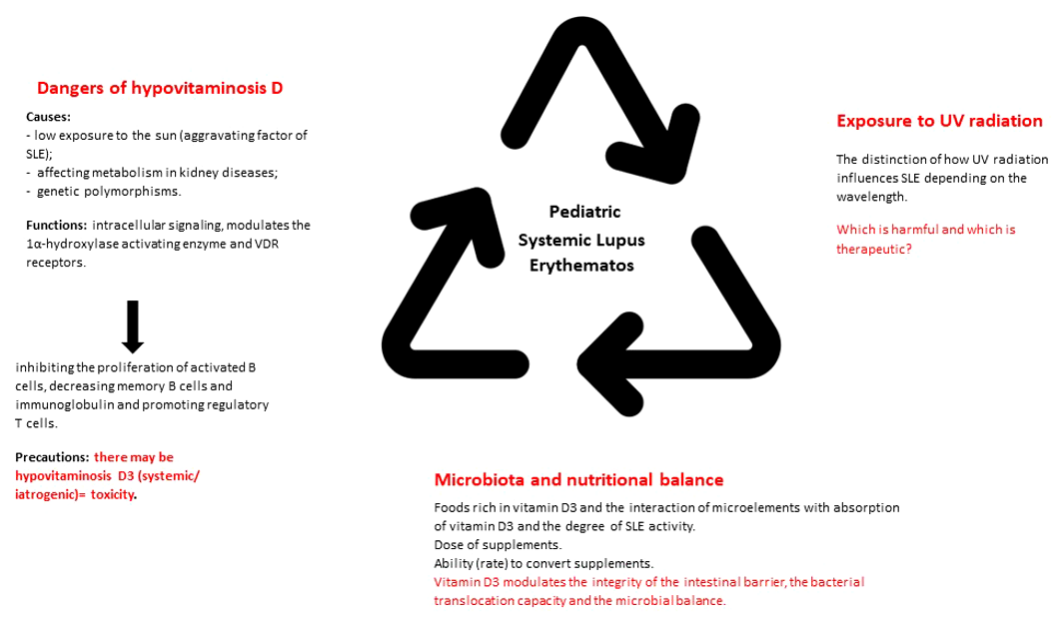
Main perspectives in the vitamin D - pSLE interrelationship.

In addition to the cardio-pulmonary investigations, we must take into account the evaluation of the neuro-psychic and renal function. In this sense, we can use the objective clinical examination, lumbar puncture with cerebrospinal fluid analysis, electroencephalogram, ophthalmological consultation, nerve conduction study or magnetic resonance imaging. The goal is to rule out an intracranial infection or neurocognitive impairment, conditions that can be frequently associated with seizures or psychosis (18, 20). Pathognomonic for kidney damage is proteinuria. This must be differentiated from orthostatic proteinuria. The objective of proteinuria, doubled by the change in the glomerular filtration rate, requires the consideration of a diagnostic renal biopsy (21). Besides these, lupus nephropathy is frequently accompanied by dyslipidemia (increase in total cholesterol, triglycerides and low/very low-density lipoproteins). Therefore, we emphasize the need for appropriate follow-up and management of lipid disorders in pediatric age (22). Next, **Figure 1** brings together the most important directions in the understanding, diagnosis and management of pSLE, from the perspective of vitamin D3.

## 4 Pathogenesis of pSLE from the perspective of hypovitaminosis D

Located on the boundary between a fat-soluble vitamin and a hormone, vitamin D, included in the class called “calciferols”, is found in the body in various forms, depending on the stage of metabolism. In order of absorption, we list ergocalciferol (vitamin D2 – supplements and fortified foods), cholecalciferol (vitamin D3 – the main food form) and, above the latter, 25(OH)D – main metabolite produced in the liver (23). Food sources rich in vitamin D3 are fatty fish (salmon, tuna, sardines, swordfish), cod liver oil, egg yolks, mushrooms, beef liver and fortified foods (cereals, milk, cheese) (7). Randomized studies place the optimal dose of supplementation among children and adolescents as being between 10-50 μg/day, this representing the interval in which maximum benefits are obtained (promotion of bone health), with minimal risks of harmful effects (24). Regarding the conversion potential of additional vitamin D3 intake, it is estimated that 100 IU has the ability to increase serum 25(OH)D by 1 ng/ml (2,5 nmol/l) (25).

As we stated previously, the source of vitamin D3 can be found in the body’s own production, at the skin level, under the influence of ultraviolet light type B (UV-B) or in exogenous, dietary intake. In the first situation, under the influence of UV-B rays, pro-vitamin D3 located in the plasma membrane of epithelial cells is converted to pre-vitamin D3 and then to vitamin D3. The latter passes into the extracellular space, where it binds to the binding protein, being transported to the liver for the purpose of its hydroxylation to 25(OH)D, the final metabolism product of this pathway. It is estimated that exposure in a bathing suit for about 15 minutes in the sun, in the middle of summer, can lead to the production of 10,000 to 20,000 IU of vitamin D3 in the case of fair-skinned people (7, 25). Regarding the exogenous intake, vitamin D3 undergoes a double hydroxylation process. After intestinal absorption and transport to the liver, the first stage of metabolism carried out under the control of cytochrome P450 2 R1 (*Cyp2R1*), the resulting form (25(OH)D) continues its course linked to the binding protein to the kidneys, where it undergoes a new process of hydroxylation regulated by *Cyp27B1*, reaching the form 1,25-dihydroxyvitamin D (1,25(OH)2D). 1,25(OH)2D is the most active form of vitamin D3, being found in all cells with specific receptors. The disadvantage of dosing this form lies in the reduced half-life in the absence of an alteration of renal function, an aspect that interferes with the optimal assessment of the nutritional status. In this sense, the dosage of the 25(OH)D form is encouraged, which has a longer half-life and thus a more accurate correlation with the actual level in the body (7, 13, 26).

The toxicity of vitamin D3 has been intensively studied over the years, both in animal models and through anecdotal studies. Currently, the metabolite responsible for the development of toxicity is not known exactly, with all of this emphasizing the importance of maintaining 25(OH)D values below the upper limit of the normal range (250 nmol/L), although clinical symptoms appear at values above 750 nmol/L (27). Another cause of toxicity is represented by the excessive production of 1,25(OH)2D (granulomatous diseases, lymphomas) or its reduced degradation in idiopathic infantile hypercalcemia. Whatever the cause, it is vital to distinguish the signs of hypervitaminosis, namely confusion, apathy, recurrent vomiting, abdominal pain, polyuria, polydipsia and dehydration (28).

The involvement of vitamin D3 in intracellular signaling is well known. This function can modulate the innate immune response, an aspect encountered in various pathologies, among which we mention autoimmune diseases precipitated by exogenous factors, respiratory diseases (e.g., tuberculosis) or others. Thus, it seems that monocytes bind the stain antigen to a toll-type receptor, in a manner dependent on the concentration of 25(OH)D, ultimately leading to the initiation of a cascade with a role in defense. The genes involved in this pathogenic cascade are 1α hydroxylase (*Cyp27*) and the vitamin D3 receptor (VDR), their activity being linked to the level of 25(OH)D (29). In this sense, we consider it important to discuss the correlation between the level of magnesium and the results of the dosages of the three forms of vitamin D. This interaction can be partially explained by the hypothesis of magnesium dependence as an enzymatic cofactor in synthesizing and metabolizing vitamin D3 (30). In the case of autoimmunity, vitamin D3 exerts its function through the 1α-hydroxylase activation enzyme and VDR receptors, located on the surface of all cells of the immune system (dendritic cells, macrophages, T and B cells). The result is the suppression of inflammation by inhibiting the proliferation of activated B cells, the decrease of memory B cells and immunoglobulin, doubled by the promotion of regulatory T cells. This mechanism may represent the correlation between vitamin D3 deficiency and SLE. Its action on dendritic cells alters the metabolic profile through intracellular signaling. A tolerogenic phenotype characterized by inhibiting the maturation and activation of dendritic cells is therefore induced, stimulating the secretion of interleukins (IL) -10 with an anti-inflammatory role. At the same time, there is a stimulation of the production of regulatory T cells and a suppression of interferon (IFN) - alpha, IL-12, IL-23 and T helper lymphocytes (Th) 1, 17 and 21 (13, 26, 31–33). It is also worth mentioning the possible polymorphism registered in the case of VDR receptors, which may present a causal association with the increase in the incidence of autoimmune diseases (34). The relationship between the intestinal microbiome and multiple systemic pathologies such as atopy (asthma), autoimmunity (SLE, celiac disease) or even post-infectious irritable bowel syndrome is well known. In the case of SLE, the presence of autoimmunity is marked at the intestinal level mainly by a low *Firmicutes - Bacteroidetes* ratio, together with an increase in *Prevotella, Rhodococcus, Eggerthella* and *Klebsiella* (35–39). Vitamin D3 also exerts effects on the integrity of the intestinal barrier, the bacterial translocation capacity and the microbial balance, thus revealing another way through which it can mediate interactions with the host’s immune system, imprinting the pathogenesis of SLE (35).

**Figure 2:**
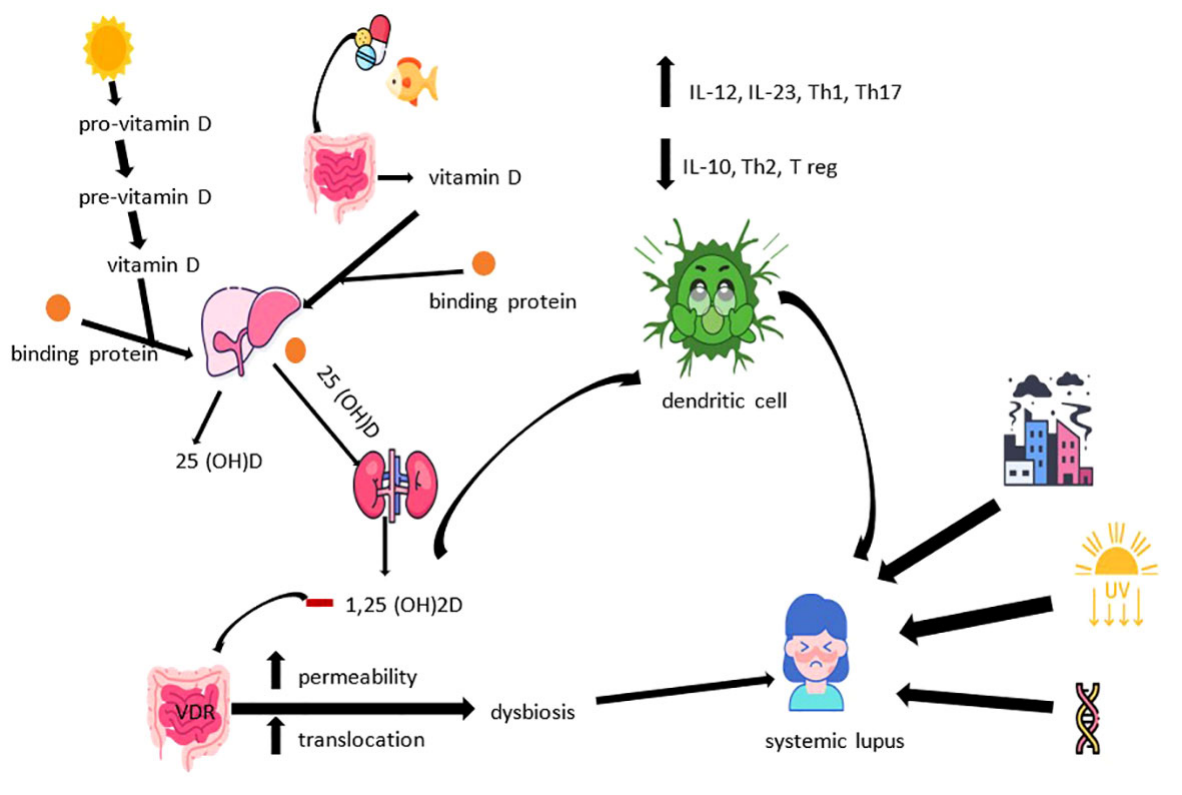
The involvement of vitamin D in the pathogenesis of SLE.

Thus, bringing together the specified data with those regarding aspects related to vitamin D3, we come to the conclusion that the pathogenesis of SLE is multifactorial. Individual, non-modifiable risk factors (genetic predisposition, birth weight, gender risk), as well as environmental factors compete for its induction. Among the latter we note exposure to ultraviolet radiation, solvents, pesticides, silica dust, drugs, oral contraceptives, infections (e.g., Epstein-Barr), certain vaccines, smoking, black tea, caffeine or stress. At the same time, alcohol consumption seems to have an inverse causal relationship with SLE, in part due to the potential to counteract systemic inflammation by reducing immuno-reactivity and suppressing the production of pro-inflammatory cytokines (tumor necrosis factor -TNF-, IL-6, IL- 8) (31, 40–42). The physiopathological cascade of SLE brings together disturbances in both innate and adaptive immunity. The main consequences of the involvement of the two are the increase of the type I IFN response, the disruption of immune tolerance with imbalances of the Th1/Th2 ratio and the dysregulation of B cells, the increase in the synthesis of autoantibodies, the decrease in the clearance rate of apoptotic cells, hypocomplementemia, the formation of immune complexes and their deposition, the inappropriate activation of neutrophils accompanied by the increase of proteases and reactive oxygen species. All of which lead to a chronic inflammatory state with multisystemic damage (e.g., skin, kidneys, joints, nervous system, cardio-vascular, respiratory) consecutive (8, 43, 44). Finally, **Figure 2** illustrates the manner in which the metabolic dynamics of vitamin D3 influence the pathogenesis of SLE.

## 5 Immunology of exposure to UV radiation

A hot point in the SLE debate is represented by the strong involvement of the sun, through UV radiation. In addition to the beneficial effects in vitamin D3 metabolism, the sun affects the quality of life of patients due to the characteristic photosensitive pattern. Thus, there is an increased need for photoprotection to avoid scaffolding caused by exposure. The interaction between the radiation and the host is directed through immune-stomatal cellular circuits (45, 46). It should be noted that these radiations are divided by wavelength (short/long). Unlike short radiations (UVB) that are harmful in SLE, current data in the specialized literature attests to the possible involvement of those with long wavelengths (UVA) in the therapeutic protocol. Low doses of UVA1 appear to reduce disease activity, along with fading of clinical symptoms and skin manifestations. The mode of action is represented by the generation of singlet oxygen, inhibition of B-cell activity, counteracting the suppression of cell-mediated immunity, modulation of apoptosis by promoting the clearance of apoptotic bodies, and slowing down the appearance of SLE-specific epigenetic changes (47–49). At the opposite pole, harmful effects caused by UVB radiation include potentiation of apoptosis, necrosis and chemokine production. These changes were found to be dependent on the interaction between apoptosis-stimulating factor (Fas) and its characteristic ligand (Fas-L). The consequence is stimulation of the immune system by activation of T cells, dendritic cells and increased IFN, simultaneously with a decrease in regulatory T cells (50–52). At the epithelial level, an increase in Ro52 expression is observed (53). Therefore, sunscreen products must be an indispensable accessory among SLE patients. Their purpose is to reduce the risk of escalating symptoms upon contact with ubiquitous UV radiation (54).

Considering the general data specified previously, one of the current concerns is represented by the possibility of affecting patients with SLE due to exposure to low, repeated doses of UVB or UVA2. They can be associated with chronic exposure to indoor halogen, incandescent or fluorescent lamps. Skin lesions were also identified in the case of using lamps from dental/surgery offices or UV lamps for cosmetic purposes (manicure). We therefore recommend bulbs with a glass cover/filter, standard manicure or, in case of non-compliance, photoprotection with sun protection factor and protective gloves (55, 56). Also, additional attention is given to the elucidation of the seasonal distribution curve of SLE cases. As we stated in the previous chapters, it is more frequently diagnosed in the cold season, being often accompanied by a low level of 25(OH)D. At the same time, the hypothesis of the overlap of its party over the consequences of increased exposure to UV radiation during the summer was launched. The data from the literature attests at the moment only the lack of correlation between the peak of the diagnosed cases and the average humidity, doubled by an inverse correlation between them and the average temperature (57).

## 6 Role of microbial and nutritional balance

### 6.1 Diet and microbiota

SLE is a multifactorial disease, whose activity can be correlated with prognostic/nutritional risk indices. The main factors involved are an unhealthy diet high in carbohydrates and low in fat (58–60). They precipitate excess weight and nutritional deficits (61). In this way, dietary patterns designed to counteract the chronic inflammation and immune impairment that are the basis of the pathophysiology of SLE can be outlined. Key points are aimed at regulating fat mass, intestinal bacterial balance and antioxidants. The optimal approach includes a diet low in sodium, balanced in iodine, potassium, magnesium, folic acid, low in protein (below 0.6 g/kg/day) and high in fiber, oils, polyunsaturated fatty acids, vitamins (A, B, C, D, E), minerals, flavonoids (lycopene, apigenin) and polyphenols (Mediterranean diet) (62–66). Curcumin (120 mg-3 g/day) can be added for its anti-inflammatory properties, with proven benefits in ulcerative colitis, rheumatoid arthritis or psoriasis (67).

The food components and the method of preparation regulate the balance of the internal environment, intestinal transit and absorption, thereby modulating the risk of atopy, autoimmunity and *Bacteroidetes-Firmicutes/Proteobacteria* balance. Functionally, vitamin A, B9, short-chain fatty acids and omega 3 fatty acids have anti-inflammatory, antioxidant and cardioprotective effects, while omega 6 and 9 fatty acids appear to improve intestinal cell junctions (64, 65, 68–72). The implication of caloric restriction and intermittent fasting is another research topic. Unlike fasting, where beneficial effects have not been identified, caloric restriction seems to positively influence SLE morbidity and mortality (31, 64, 73–75).

Regarding the microbiota, it is important to know that it is in a perpetual change since the neonatal period, influencing the evolutionary course and the response to the treatment of various pathologies through gut-vital organ axes (76–82). Another factor inducing intestinal inflammation and dysbiosis is stress (83). Summarizing, intestinal dysbiosis is characterized by the inversion of the *Firmicutes/Bacteroides* ratio, the decrease of *Lactobacillaceae* and the increase of *Lachnospiracea* and Proteobacteria (*Sphingomonas*) due to the alteration of the barrier of the upper digestive system. Diet, supplementation with retinoic acid and probiotics, prebiotics or symbiotics have proven effective in restoring homeostasis. The possible mechanisms of action are the modulation of the expression of chemokine receptors in dendritic cells and the decrease of Th17 lymphocytes, IL-17, Toll-like receptors (TLR) -4, TLR-5, TLR-7 and TLR-9, together with the favoring of regulatory T cells. However, the administration of Lactobacillus can also be harmful, depending on the environmental and individual characteristics of the patient (63, 64, 84–88). The SLE activity estimated by the Systemic Lupus Erythematosus Disease Activity Index (SLEDAI) activity score seems to be correlated with the variable abundance of fecal and salivary bacteria. At the same time, it differs depending on the levels of the C3 and C4 factions of the complement (89). Studies in the specialized literature confirm both the involvement of intestinal and oral dysbiosis in dictating the risk of manifest autoimmunity, as well as the role of diet components (e.g., tryptophan) in modulating the intestinal microbiota in healthy people and SLE patients (90–93).

### 6.2 Nutritional dosages

In addition to clinical and paraclinical diagnosis, as in any disturbance of the homeostasis of the internal environment, the dosages of nutritional components and the highlighting of dietary deficiencies or, on the contrary, of excess, have also proven useful in the understanding and management of SLE cases. To briefly exemplify the influence of nutritional deficiencies on the body’s balance, we discuss the case presented by Mishra VA. et al. which was manifested by intermittent low fever, fatigue, anorexia, muscle weakness, anemia and severe thrombocytopenia, without localized symptoms characteristic of any system. The general investigations were within the limits of normal, being objectified the deficiency of 25(OH)D and vitamin B12, a rare but known cause of the manifestations stated above which, once treated, was accompanied by the remission of the symptoms, without further relapses (94).

Considering this brief example, we would like to specify that even among patients with SLE, the most important nutrients when it comes to dosage are vitamins. Due to the multiple roles, it plays in the body, starting from the regulation of chronic inflammation (cytokines and inflammatory markers), anti-inflammatory effects, directing the maturation and regulation of immune cells, decreasing the production of antibodies and oxidative stress, but also marking the risk of cardiovascular events, B vitamins (B1, B2, B6, B9, B12) and vitamins E, C and A play a major role in dictating the biological changes of any SLE patient. Thus, the priority of supplementation is emphasized when they are in deficit (64). Regarding the markers that can be dosed in order to evaluate the nutritional status and the cardiovascular risk, Salomão RG. et al. report homocysteine, tumor necrosis factor alpha (α-TNF), high-sensitivity CRP and folic acid levels, along with dyslipidemias, as having a relevant association (95). The first parameter presented a confirmed association with pSLE, but not with renal damage, disease activity, overweight or short stature (96).

The electrolytes are also necessary to be evaluated, since sodium and potassium compete to modulate the Th17/T regulatory balance, inflammation, the level of anti-dsDNA antibodies and the complement fractions (C3 and C4) and consequently the individualization of the diet according to them may have benefits on symptom control (64). Along with these, other essential trace elements (iron, copper, zinc, selenium) have proven their necessity in the differentiation, activation and functioning of immune cells. Iron in particular can direct CD4 T cells to a pro-inflammatory phenotype, although regulatory T lymphocytes have been shown to be resistant to low iron intake (44).

## 7 Is hypovitaminosis D an alternative therapeutic target in pSLE?

Vitamin D3 deficiency is among the most frequent and well-known nutritional deficiencies identified in the cases of children with SLE. Reinforcing what we previously stated, Stagi S. et al. bring to light the multiple implications of vitamin D3 metabolism in the activity of systemic lupus erythematosus and its associated complications. The novelty presented by the working group is the possible association of a genetic mutation that predisposes to hypovitaminosis D in the non-SLE population of European origin, a previously debated aspect. In accordance with their invitation to continue the debate, we will review the main evidence found in the literature regarding the influence of hypovitaminosis D during pSLE, but also the impact of its sublimation (97).

Rosiles VH. et al. reports with the help of a study carried out on a group of 153 children and adolescents, low levels of 25(OH)D in the group of patients with pSLE compared to the control group (18 ng/ml versus 22.3 ng/ml) (98). Similar results were obtained by Comak. et al. and Cheng. et al. The peculiarity of their studies, although performed on a relatively small group of children, resided in the objective of a significant negative correlation between serum 25(OH)D concentrations and SLE activity scores. Added to this is the inverse correlation between the administration of corticotherapy and the serum level of 25(OH)D (99, 100). Other objectified correlations were represented by the positive association between increased values of IFN-γ and IL-17 and pSLE (101). Next, AlSaleem A. et al. underlines that, after a 3-month treatment with vitamin D3 and calcium, an improvement in the activity score and specific autoimmunity markers was observed. Consequently, they postulated the positive correlation between the intense activity of the disease in childhood and hypovitaminosis D (102). In addition to the already stated multiple roles of vitamin D3 in maintaining the body’s homeostasis, Sultana N. et al. underlines the importance of investigating its serum levels, together with supplementation until the necessary optimal values are obtained. This desire resides from the objectification of the correlation between the hypoperfusion of the cognitive areas of the brain found, the neuro-psychic manifestations characteristic of lupus and hypovitaminosis D (103).

Thus, vitamin D3 deficiency cannot be neglected due to its involvement in dictating the susceptibility and severity of SLE. In order to understand which of the two disturbances (hypovitaminosis D and SLE) is the first to appear, we consider it appropriate to discuss the identification of a pattern of occurrence of autoimmune diseases, including SLE, represented by a peak of the incident in April and a minimum in October. This predominantly seasonal distribution of newly diagnosed cases rather incriminates 25(OH)D deficiency as a risk factor in SLE, rather than as a consequence of the condition (104). However, the data in the literature are far from unanimous. Ding Y. et al. they recognize the inconsistency of the relationship vitamin D3 - SLE depending on race, geographical and seasonal factors, but they place vitamin D3 among the negative reactants of the acute phase, arguing that hypovitaminosis occurs as an effect of acute inflammation and denying its involvement in increasing the risk of SLE (105). In addition to this, hypovitaminosis D caused by polymorphisms of genes involved in its transport, binding and metabolism (e.g., *CYP24A1*) has been shown to be correlated with increased susceptibility to SLE among relatives of subjects already diagnosed positively (106).

Lin TC. et al. concluded, by following a cohort of patients in the active and inactive periods of the disease, that pSLE and 25(OH)D levels are inversely correlated (107). Regarding the severity of SLE, it is considered that the key pillar in its dictation is represented by IFN-α, an inducer of the differentiation of monocytes towards dendritic cells, whose balance is influenced in the sense of its inhibition by vitamin D3. Magro R. et al. We support this hypothesis by the positive results (regarding the activity of the disease and the degree of fatigue of the patients) obtained when supplementing with 25(OH)D in deficient/ineffective cases, results that were attributed to the inhibition of the expression of the interferon signature gene (108–110). Similar effects were observed by Lima GL. et al. when supplementing subjects with pSLE for 24 weeks with cholecalciferol (111).

In the same direction, Irfan SA. et col. attests, with the help of a meta-analysis that included studies focused on adult populations, the positive effect of vitamin D3 supplementation on the disease activity score and the serum level of the C3 fraction of the complement, but not on the C4 fraction and anti-dsDNA antibodies (112). Also, the low level of 25(OH)D correlates with osteoporosis, fatigue and potentiation of cardiovascular risk factors (decrease in renin activity, increase in peripheral resistance to insulin, reduced immunomodulatory effects, alteration endothelial function and increased coronary artery calcification). An increased association of hypovitaminosis D in adults and stroke, myocardial infarction, diabetes, hypertensive heart disease, obesity and dyslipidemia was demonstrated. All these bring a negative addition to the morbidity induced by SLE. The involvement of hypovitaminosis D in dictating an additional increased risk of infections or malignancies remains open to study. The optimal doses for supplementation must be adapted depending on the basic level of 25-hydroxyvitamin D, BMI, the doses of steroids used chronically, the tolerability of the patients and the presence of other risk factors. A target 25(OH)D level of at least 30 ng/ml is desired (113–117).

In accordance with the above, **Table 2** facilitates an overview of the prevalence of 25(OH)D deficiency in SLE patients, its correlation with disease activity and biological markers, and the effects of its supplementation.

**Table 2:** Studies on the impact of hypovitaminosis D in SLE and the effect of supplementation.

Study	Design	N	Purpose	Investigations/Method	Result	Comments
Hamza RT. et al. (6)	CC	120	The relationship between low 25(OH)D levels and SLE activity	Serum 25(OH)D test by ELISA; Calcium, phosphorus, parathyroid hormone and ALP dosage; Estimation of ANA and anti-dsDNA antibodies;	73.30% of patients with SLE had a low level of vitamin D3 (13% deficiency, 60% insufficiency). The level of 25(OH)D was not correlated with age and duration of the disease.	A correlation has been identified between low vitamin D3 levels in SLE patients and increased disease activity or photosensitivity.
Robinson AB. et al. (14)	CC	58	Determining the correlation between 25(OH)D level and disease activity/proteinuria	25(OH)D dosing; urinary vitamin D3 binding protein/creatinine ratio; estimation of urinary protein/creatinine ratio; albuminuria dosage;	The positive correlation between the deficiency of vitamin D3 in SLE and the present proteinuria was highlighted.	Children with severe vitamin D3 deficiency had kidney disease, thus not being able to calculate the odds ratio of kidney disease - severe vitamin D3 deficiency.
Rosiles VH. et al. (98)	CC	153	Determination of 25(OH)D concentration in patients with SLE	Dosage of vitamin D3, parathyroid hormone, calcium, phosphorus, ALP;	The concentrations of 25(OH)D were lower (18.9 ng/ml) in the SLE group compared to (23.6 ng/ml) among healthy patients. 10 patients with SLE had vitamin D3 levels <12 ng/ml.	Patients receiving vitamin D3 supplements and those in the control group who had relatives with a history of SLE were excluded from the study.
AlSaleem A. et al. (102)	Cs	28	25(OH)D status in children with pSLE and the effects of doubled calcium supplementation	Samples collected included total 25-OH vitamin D, bone profile, parathyroid hormone, erythrocyte sedimentation rate (ESR), urine for protein/creatinine ratio and calcium/creatinine ratio, complement (C3, C4) levels, anti-dsDNA, ANA levels, and bone markers (osteocalcin, B-telopeptide). Disease activity was assessed by the SLEDAI score.	Levels of vitamin D3 was correlated inversely with ANA, anti-dsDNA titers, and SLEDAI scores. After 3 months of treatment: 20 patients showed improvement in their 25-OH vitamin D levels, 17 showed improvement in ANA, anti-dsDNA titers and complement levels as well as SLEDAI scores and 9/18 with high protein/creatinine ratio showed significant reduction in proteinuria.	There were no adverse effects reported among the patients taking vitamin D3 during the period of observation.
Lin TC. et al. (107)	CCh	70	Targeting the correlation between SLE activity and 25(OH)D level	Estimation of urinary sediment and proteinuria; Dosage of vitamin D3 level; blood count, creatinine, complement fractions, autoantibodies;	Vitamin D3 deficiency was significantly higher in the active period, compared to the inactive period of the pathology.	The link between active lupus nephritis and a more severe hypovitaminosis D was reiterated.
Lima GL. et al. (111)	R	40	Evaluation of the intake brought by 25(OH)D supplementation in patients with pSLE	Administration of cholecalciferol 50,000 IU/week or placebo, for 24 weeks.	Considerable improvement of SLEDAI and ECLAM in the group with vitamin D3 compared to placebo, at the end of the period. In parallel, fatigue was reduced and social integration improved.	Tolerance and adherence to therapy were good, without serious adverse events.
Arshad A. et al. (114)	CC	98	Assessment of prevalence of 25(OH)D deficiency in Pakistani patients with SLE and correlation with disease severity	Disease activity was also monitored by complement (C3, C4) levels and anti-dsDNA.	Vitamin D3 deficiency is an extremely common event in SLE patients (2/3 being deficient and >1/2 being severely deficient). There was a strong association between 25(OH)D deficiency and disease activity.	Other studies have shown that the prevalence of the deficiency varies depending on a number of factors, such as environmental, geographic and genetic factors.
Attar SM. et al. (115)	CCh	95	Studying the relationship between 25(OH)D and disease activity in patients with SLE	General evaluation, disease duration and activity, skin manifestations, history of lupus nephritis, liver damage, malabsorption, long-term use of steroids >60 days, steroid-sparing agents, anticonvulsants, anti-dsDNA serum levels, complement and vitamin D3.	No correlation was detected between vitamin D3 and SLEDAI score. There was a negative correlation between vitamin D3 and anti-dsDNA, doubled by a positive correlation with C4.	Azathioprine, low C3 and C4, and active disease have been associated with 25(OH)D deficiency.
Fiblia F. et al. (116)	R	60	Impact of cholecalciferol supplementation on disease activity	The patients were divided into two groups, study and placebo. Cholecalciferol 5000 IU/day was administered for 12 weeks. The severity of the disease was evaluated using the MEX-SLEDAI score.	Increased 25(OH)D levels and improved disease activity were observed.	No significant improvement in the quality of life of SLE patients was obtained
Zheng R. et al. (117)	M	490	Efficacy of 25(OH)D supplementation in patients with systemic lupus erythematosus	Five randomized controlled trials were included.	Vitamin D supplementation is effective in increasing serum 25(OH)D levels, can improve fatigue, and is well tolerated in patients with SLE	Inconveniently, this does not appear to have significant effects in decreasing anti-dsDNA positivity and disease activity

CC, case-control; CCh, case-cohort; Cs, cross-sectional; R, randomized; M, Meta-analysis; N, number; SLEDAI, Systemic lupus erythematosus disease activity index; ECLAM, European Lupus Activity Measurement Consensus; ELISA, enzyme-linked immunosorbent assay; ALP, alkaline phosphatase; anti-dsDNA, anti-double-stranded deoxyribonucleic acid; ANA, antinuclear antibody.

## 8 Lupus and vitamin D3 in the pandemic

Being a condition with multisystemic manifestations, the recent Covid-19 pandemic has attracted an increase in the incidence and severity of a multitude of pathologies. Among these, as is to be expected from the perspective of the physiopathogenic basis aimed at disturbances of the immune system, there are autoimmune diseases. De Belo IA. et al. underlines the ability of SARS-CoV-2 infection to trigger a pro-inflammatory state, accompanied by an increase in the activity of inflammatory cytokines and the consequent triggering of an aberrant immune response. Their hypothesis is based on the presentation of the case of an 11-year-old girl, previously healthy, who after the first wave of the pandemic developed arthralgias, pericarditis, pleurisy and ANA and anti dsDNA positivity, accompanied by a decrease in the level of the C3 fraction of the complement (positive diagnostic criteria for SLE). In the evolution, the clinical symptoms and the biological parameters remitted after corticotherapy and administration of Hydroxychloroquine (118). A similar case, this time in a 1-and-a-half-year-old girl, was reported by Sobh A. et al. (119). Although the two cases impress with the extent of the systemic manifestations, it should be specified that the initial manifestation of the condition was centered on the skin-articular component (arthralgias, oral ulcerations, erythemato-maculopapular rash on the face and body with areas of hyperpigmentation, vasculitic rash and edema in both hands and feet). On the opposite side, Domínguez-Rojas J. et al. reports the case of an 11-year-old boy, whose first diagnostic hypothesis was multiorgan failure syndrome in the context of Covid-19 infection. Biological investigations refuted the infection, and the analysis of the differential diagnosis possibilities led to the incrimination of the macrophage activation syndrome that appeared in the context of SLE as the cause of the multiorgan failure Covid-19-like, in terms of the response to the specific therapy being satisfactory (120). The relationship between the two entities therefore seems to be bidirectional, during the Covid-19 pandemic, noting both the exacerbation of already diagnosed pSLE cases and the appearance of new cases, as well as the increased risk of these patients to develop viral infections with an accentuated severity compared to that of the general population. Also, although the vaccine seems to be safe in the pediatric population with autoimmunities, the question arises as to how immunosuppressive therapy can influence its effectiveness (121, 122). Another point of intersection of the two entities is represented by the changes found in the gastrointestinal tract, which have been shown to be similar between patients with Covid-19 and those with SLE. It is therefore important to know and adequately treat intestinal dysbiosis, given the low costs and risks of counteracting it, which are accompanied by favorable effects in the evolutionary course of the pathology, but also by the possible decrease in the risk of interhuman transmission (123).

Vitamin D3 was also essential during the pandemic, its level being influenced and influencing clinical manifestations and the risk of developing Covid-19 (124–128). One of the causes responsible for the exacerbation of 25(OH)D deficiency in children and adolescents during the pandemic period may be the imposition of isolation restrictions, felt more strongly among children over 6 years old (129, 130). In this sense, Mercola J. et al. pencils, with the help of the existing data in the literature, the main means of action of 25(OH)D deficiency that dictate the risk, severity and mortality in SARS-CoV-2 infection. Among these we note the stimulation of antiviral mechanisms (e.g., antimicrobial peptides), increases the concentrations of angiotensin-converting enzyme 2 and reduces the risk of death caused by acute respiratory distress syndrome, pro-inflammatory cytokines, the risk of endothelial dysfunction, metalloproteinase-9 concentrations and increasing the level of bradykinin (131). Thus, the severe deficiency of 25(OH)D predisposes children to cardiovascular damage and disturbances of the immune response, which potentiates the severity of the multisystemic inflammatory syndrome determined by Covid-19 (132). Although there are still no regulations regarding the treatment with vitamin D3, we conclude that its existence in adequate amounts can have a prophylactic, immunoregulatory, but also neuroprotective role (it regulates neurotrophins, promotes the migration and differentiation of oligodendrocyte precursors and increases neurotransmission following the improvement of neuronal myelination) in the case of subjects with Covid-19 (133). Another nutritional deficiency found among pediatric patients positive for SARS-CoV-2 was that of zinc (134).

Cumulating the exposed data, we observe a pronounced link between SARS-CoV-2 infection, vitamin D3 deficiency, doubled by zinc deficiency, and the development/exacerbation of pSLE. We also reiterate the strong link between vitamin D3 deficiency and SLE manifestations. Therefore, it is vital to outline some management and adequate care programs for these pathologies reflected in the pediatric sphere. These must be focused both on adequate treatment, supplementing nutritional deficiencies and counteracting associated comorbidities, as well as on the integration of patients in psychotherapy and physical exercise programs, all with the ultimate goal of increasing the quality of life (135, 136).

## 9 Conclusions

This review updates the data on the pathophysiological and therapeutic implications of vitamin D3 in the dynamics of pediatric systemic lupus erythematosus. The narrative exposition can be summed up by the oft-used phrase “we are what we eat”. We have therefore presented general aspects related to pSLE, risk factors and immunological mechanisms through which the metabolism of vitamin D3 intervenes in the balance of the internal environment, unmasking or aggravating the symptoms of lupus. Among the predisposing factors, we delineated the unhealthy lifestyle and nutritional deficiencies. Both the effects of excesses and deficiencies of these constituents have been debated. In addition, the interaction between pharmacological substances - vitamin D3 and the weight of the recent Covid-19 pandemic in stimulating the advancement of research in the field by establishing a bidirectional link between the two pathologies have not been neglected. Finally, we consider it appropriate to initiate new studies focused on the comparative analysis of the beneficial versus harmful effect of sun exposure in the pediatric population with lupus and beyond. Due to its duality, the sun is both an aggravating factor of SLE, as well as a necessary factor in the formation of vitamin D3, an important constituent in the immune, inflammatory and microbial balance.

## Author contributions

VL: Conceptualization, Writing – review & editing. AL: Methodology, Writing – original draft. JE: Conceptualization, Writing – original draft. IS: Writing – review & editing, Methodology. GS: Methodology, Writing – original draft. II: Investigation, Writing – original draft. AA: Investigation, Writing – original draft. CD: Visualization, Writing – review & editing. AK: Visualization, Writing – review & editing. RB: Investigation, Writing – original draft. DS: Writing – review & editing. NR: Writing – review & editing, Supervision. SF: Project administration, Writing – review & editing.
